# Single-cell transcriptional atlas of tumor-associated macrophages in breast cancer

**DOI:** 10.1186/s13058-024-01887-6

**Published:** 2024-09-04

**Authors:** Yupeng Zhang, Fan Zhong, Lei Liu

**Affiliations:** 1 Institutes of Biomedical Sciences, Fudan University, Shanghai, 200032 China; 2 Intelligent Medicine Institute, Shanghai Medical College, Fudan University, Shanghai, 200032 China

**Keywords:** TAMs, Tumor immunity, Single-cell transcriptome, Cellular atlas, Cell-cell communication

## Abstract

**Background:**

The internal heterogeneity of breast cancer, notably the tumor microenvironment (TME) consisting of malignant and non-malignant cells, has been extensively explored in recent years. The cells in this complex cellular ecosystem activate or suppress tumor immunity through phenotypic changes, secretion of metabolites and cell-cell communication networks. Macrophages, as the most abundant immune cells within the TME, are recruited by malignant cells and undergo phenotypic remodeling. Tumor-associated macrophages (TAMs) exhibit a variety of subtypes and functions, playing significant roles in impacting tumor immunity. However, their precise subtype delineation and specific function remain inadequately defined.

**Methods:**

The publicly available single-cell transcriptomes of 49,141 cells from eight breast cancer patients with different molecular subtypes and stages were incorporated into our study. Unsupervised clustering and manual cell annotation were employed to accurately classify TAM subtypes. We then conducted functional analysis and constructed a developmental trajectory for TAM subtypes. Subsequently, the roles of TAM subtypes in cell-cell communication networks within the TME were explored using endothelial cells (ECs) and T cells as key nodes. Finally, analyses were repeated in another independent publish scRNA datasets to validate our findings for TAM characterization.

**Results:**

TAMs are accurately classified into 7 subtypes, displaying anti-tumor or pro-tumor roles. For the first time, we identified a new TAM subtype capable of proliferation and expansion in breast cancer-TUBA1B^+^ TAMs playing a crucial role in TAMs diversity and tumor progression. The developmental trajectory illustrates how TAMs are remodeled within the TME and undergo phenotypic and functional changes, with TUBA1B^+^ TAMs at the initial point. Notably, the predominant TAM subtypes varied across different molecular subtypes and stages of breast cancer. Additionally, our research on cell-cell communication networks shows that TAMs exert effects by directly modulating intrinsic immunity, indirectly regulating adaptive immunity through T cells, as well as influencing tumor angiogenesis and lymphangiogenesis through ECs.

**Conclusions:**

Our study establishes a precise single-cell atlas of breast cancer TAMs, shedding light on their multifaceted roles in tumor biology and providing resources for targeting TAMs in breast cancer immunotherapy.

**Supplementary Information:**

The online version contains supplementary material available at 10.1186/s13058-024-01887-6.

## Background

Breast cancer, a prevalent malignant neoplasm in females, stands as the second leading cause of oncological mortality globally among women after lung cancer [1]. The heterogeneity inherent in breast cancer has been the subject of extensive research since the 19th century [2]. Breast cancer was initially categorized into initial categorization including luminal, human epidermal growth factor receptor 2 positive (HER2^+^) and basal-like breast tumors according to histopathology, molecular subtypes and clinical features [3]. Now the exploration of its diversity has evolved into a more detailed understanding of the TME, which reveals a shift from the inter-tumor heterogeneity the spatiotemporal intra-tumor heterogeneity. The TME constitutes a complex ecosystem, characterized by the dynamic interplay between malignant tumor cells and a range of immune and non-immune cells [4]. Within the TME, several factors converge to influence tumor development and progression, which include the secretion levels of nitric oxide and reactive oxygen species (ROS), the persistent presence of chronic inflammation, the extracellular matrix (ECM) serving as a critical scaffold which facilitates the targeted trafficking of proteins and cells and bidirectional interactions between malignant and non-malignant cells [5, 6].

The occurrence, progression and metastasis of tumors are inseparably linked to the remodeling of the immune microenvironment. Macrophages are integral parts of the TME, accounting for nearly half of the total mass of non-malignant cells in the TME, significantly influencing tumor progression [7]. As the tumor progresses, the complexity and heterogeneity of macrophages increase, underscored by their activation and polarization dynamics [8]. Macrophages can be divided into two types based on their polarization, namely M1 and M2 macrophages. M1 macrophages are activated through a Type I immune response by toll-like receptor ligands and Type I cytokines including tumor necrosis factor α (TNF-α) and Interferon-γ (IFN-γ) [9]. Once activated, M1 macrophages will secrete pro-inflammatory cytokines such as IFNs, interleukin-12 (IL-12) and IL-23. Due to their antigen-presenting capability and pro-inflammatory properties, they are considered anti-tumor macrophages [10]. While M2 macrophages are activated by cytokines IL-4, IL-13 and immune complexes, inducing a T helper 2 (Th2) type response and producing TGF-β and other pro-fibrotic factor, thereby harboring the potential to promote tumor progression [11]. Although this classification system is still widely used, it fails to accurately characterize the phenotypic and functional heterogeneity of macrophages within tumors. Single-cell sequencing (scRNA-seq) has provided new insights into the analysis of TAMs. Nearly all macrophage subtypes which exhibit distinct marker genes as well as vary in morphology and function within the TME co-express M1 and M2 characteristics [12]. The discovery of TAMs further reveals the heterogeneity and functional diversity of macrophages in cancer [13]. These cells are associated with angiogenesis, ECM remodeling as well as tumor growth and invasion. TAMs not only exert direct effects on cancer cells but also interact with other non-malignant cells including stromal cells and other immune cells within the TME through direct contact or paracrine signaling [14], which contribute to tumor progression, metastasis and therapeutic resistance [15]. Due to the influence of cancer cells, the chronic inflammatory environment and small molecule metabolites in reshaping the cells within the TME, TAMs exhibit spatiotemporal phenotypic and functional heterogeneity. Their heterogeneity lies not only in genomic, epigenomic, transcriptomic and intrinsic phenotypic differences in various tumor regions, but also in the temporal evolution of tumor progression processes at various tumor sites [16].

Although recent studies have characterized the TME of breast cancer [17, 18], there is still a lack of systematic research on the subtyping and functional definition of TAMs. Utilizing published scRNA-seq data from untreated primary breast cancer tissues, we conducted a detailed and biologically meaningful systematic classification of TAM subtypes, clarified their effects on tumor immunity and validated the clinical relevance. For the first time in breast cancer, we identified TUBA1B^+^ TAMs with proliferative and expansional ability, which may serve as precursors for other TAM subtypes. We focused on pinpointing differentiation events within TAMs to reveal how TAMs are reshaped and undergo phenotypic and functional changes within the TME. To explore how TAMs operate through cell-cell communication within the complex and dynamic TME, we used specific subtypes of ECs and T cells as key nodes to investigate the significant ligand-receptor pairs involved in interactions with various TAM subtypes.

This study establishes an accurately segmented single-cell atlas of breast cancer TAMs, unveiling their heterogeneous unique composition and characteristics. This comprehensive atlas offers an unprecedented resource for understanding the complexity of breast cancer TAMs and guiding the development of TAM-targeted immunotherapies for breast cancer.

## Methods

### scRNA-seq data processing

The scRNA-seq dataset (GSE167036 and GSE248288) was downloaded from the Gene Expression Omnibus (GEO). We focused on samples labeled as “tumor”. The expression matrix was processed using the Seurat package (version 4.3.0) in R (version 4.2.2). During the preprocessing stage, we conducted quality control based on sequencing depth and mitochondrial reads. Low-quality cells characterized by unique molecular identifiers (UMIs) < 400 or mitochondrial RNA > 25% were excluded. Following the quality control measure, 49,141 single cells from dataset GSE167036 were included in downstream analysis and 25,925 single cells from dataset GSE248288 were selected for validation.

### Identification of main cell types

The gene expression matrix was firstly normalized utilizing the default parameters of the Seurat package. Subsequently, the FindVariableFeatures function was employed to identify highly variable genes, followed by scaling of the gene expression matrix. The reduction in gene dimensions was achieved through principal component analysis (PCA) applied to the normalized and standardized gene expression matrix using the Seurat package. The Harmony algorithm accurately integrates single-cell data from different technology platforms and batches [19]. Considering batch effects due to the scRNA-seq data from eight independent patients, we employed RunHarmony function of the harmony package (version 1.1.0) to mitigate these effects. Cell clusters were identified using FindClusters (resolution = 1.2) in Seurat, followed by visualization of cells in a 2D space using uniform manifold approximation and projection (UMAP). In this way, unsupervised clustering clustered cells into 35 clusters (GSE167036) and 40 clusters (GSE248288). Marker genes for each cluster were identified using FindAllMarkers, requiring expression in at least 25% of cells within a cluster. To identify main cell types, supervised clustering was performed using the DatabaseImmuneCellExpressionData in the SingleR package (version 1.10.0). We then conducted direct analysis for cell type identification based on marker genes, using the results of supervised learning as a basis. This resulted in the classification of main cell types.

### Identification of subtypes

Subsets of each main cell type were extracted from the original expression matrix for further sub-clustering to elucidate subtype structures. The clustree algorithm [20] determined appropriate resolutions for biologically meaningful clustering-myeloid cells (resolution = 1.5), ECs (resolution = 1.0) and T cells (resolution = 1.2). Marker genes for each subtype were identified using FindAllMarkers and combined to ensure biological relevance of the clusters.

### Enrichment analysis

In order to conduct enrichment analysis and elucidate the functions of 7 distinct TAM clusters, we first identified differentially expressed genes (DEGs) within each of these clusters. For a gene to be considered a DEG in any cluster, it had to meet specific criteria: an adjusted *P*-value < 0.05 and log2| Fold Change| > 0.25. Subsequently, we performed enrichment analysis on the DEGs of all 7clusters. The Metascape (https://metascape.org) was utilized to annotate representative biological functions based on significant DEGs, using datasets such as Gene Ontology biological processes, KEGG pathways, Reactome and Wikipathways [21]. Additionally, in exploring biological processes related to cellular developmental trajectory, we applied the same methodology to conduct enrichment analysis on six gene clusters that exhibited significant expression changes between two distinct cell fates in order to reveal the biological changes that occur during developmental trajectory.

### Cell developmental trajectory

The analysis of cell developmental trajectory was conducted using the Monocle2 package (version 2.24.1) in an unsupervised manner, which aims to calculate the pseudotime trajectory of TAMs to further reveal the dynamic transitions in cellular states [22]. To reduce the dimension, the DDRTree algorithm was employed, and trajectory was constructed using DEGs with *Q*-value < 0.01. The starting point of the pseudotime trajectory is determined based on specific cellular states. The visualization of the trajectory was achieved through the plot_cell_trajectory function. Furthermore, selected genes were extracted using the differentialGeneTest function, and regression analysis was performed to delineate their expression patterns over pseudotime more clearly.

### Cell-cell interaction analysis

In order to explore the potential interactions between different cell types, cell-cell interaction analysis was carried out using the CellPhoneDB [23] Python package (version 3.0.0), based on the expression of known ligand-receptor pairs. Interaction between two cell types is inferred when one cell type expresses the ligand and the other expresses the corresponding receptor. “Expression” here is defined as at least 10% of the cells in a given cell type exhibiting non-zero read counts for the respective receptor or ligand genes. Enriched ligand-receptor interactions between two cellular subgroups were calculated through permutation tests. Significantly enriched ligand-receptor pairs with *P*-value < 0.05 were extracted to predict the potential interaction strengths between 7 subtypes of TAMs and ECs, as well as T cell subgroups.

### Survival analysis

In this study, we evaluated the prognostic performance of the 7 distinct subtypes of TAMs identified, using data from the TCGA BRCA dataset which included 313 patients who had not undergone endocrine therapy or chemotherapy. Following methodologies similar to those used in previous studies [24], we conducted overall survival (OS) analysis based on the expression of specific highly expressed genes in 7 types of TAMs normalized by *CD68*. For each gene, the cutoff point for grouping was determined by an automatically selected optimal threshold. Kaplan-Meier survival curves were generated and Hazard Ratio (HR) along with *P*-values from log-rank tests were computed using the online Kaplan-Meier plotter tool (https://kmplot.com). For those TAM subtypes unable to be characterized by individual genes, we employed OS analysis based on their signature gene sets.

### Generative AI in scientific writing

In order to improve readability and linguistic proficiency, artificial intelligence-assisted techniques were used. However, the final result was entirely done by the authors, who carefully edited the language to conform to the domain terminology. Consequently, we take full responsibility for the content of this study.

### Data availability

The scRNA-seq dataset (GSE167036) was downloaded from the GEO database, https://www.ncbi.nlm.nih.gov/geo/).

## Results

### A single-cell transcriptomic atlas of human breast tumor tissue

**Fig. 1:**
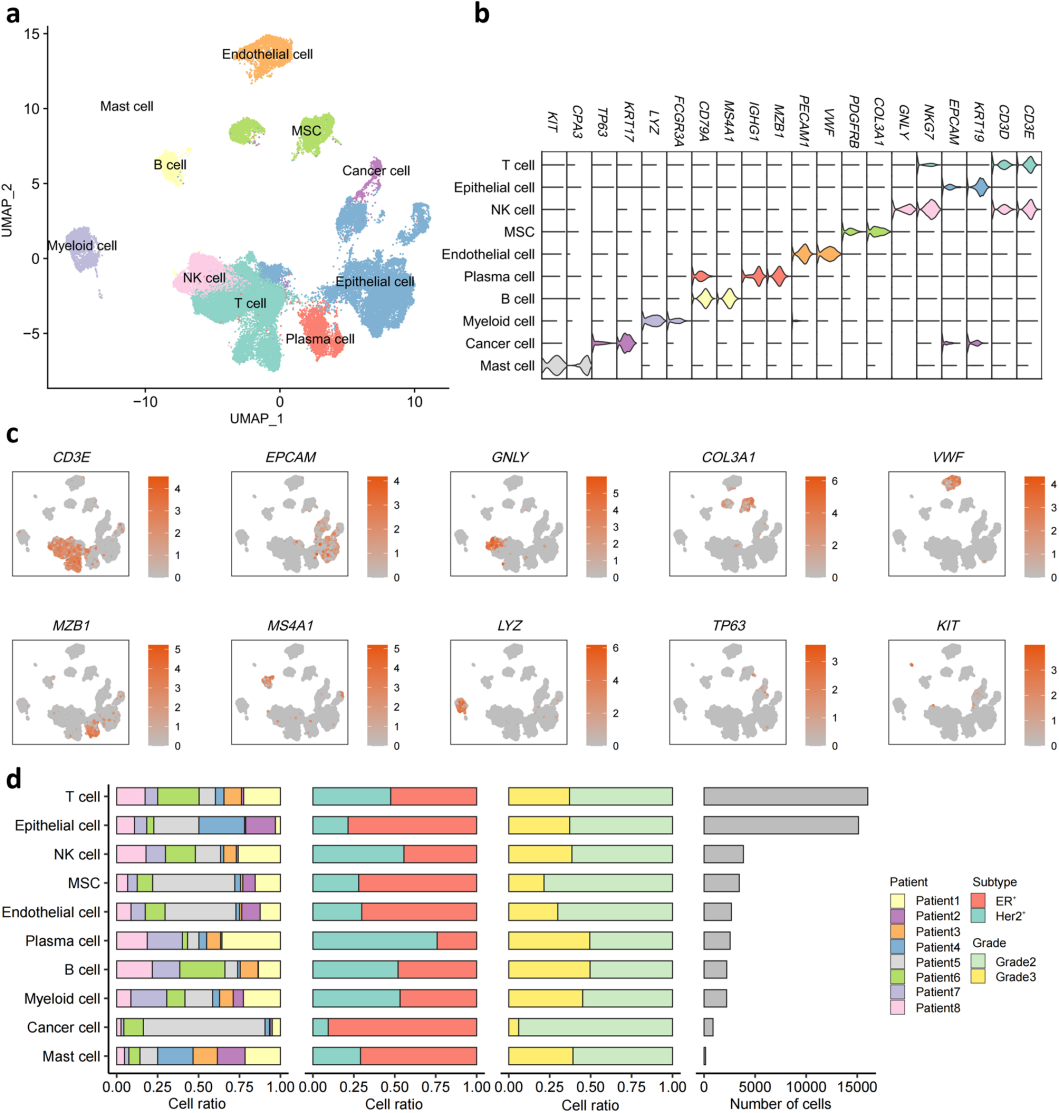
Single-cell atlas of human breast cancer. **a** UMAP plot of 49,141 cells from tumor tissue of the 8 untreated breast cancer patients, showing 10 clusters in the plot. Each cluster was shown in different color. R package harmony was used to correct batch effects and constructed one UMAP based on all tumor cells. **b** Violin plot showing the expression of selected signature genes. Colors as in **a**. **c** Expression levels of selected known marker genes across 49,141 unsorted cells illustrated in UMAP plots from tumor tissue in breast cancer patients. **d** Proportion of the 10 major cell types showing in bar plots in different donors (the first panel), subtypes (the second panel), grades (the third panel) and total cell number of each cell type (the fourth panel) are shown

Publish single-cell data from primary tumor tissues of 8 untreated patients with luminal A, luminal B and HER2^+^ subtypes of breast cancer from 10X Genomics platform were included in our study. By clustering 49,141 single cells of 8 samples in the atlas, we identified 35 clusters (Fig. S1) and labeled each cluster with its respective markers. Using UMAP clustering, we generated a 2D representation of these clusters. Based on their typical markers, all cells can be categorized into the following ten main types (Fig. 1a, b): T cells (*n* = 16,033) defined by T cell receptor (TCR) signaling mediators *CD3D* and *CD3E*, epithelial cells (*n* = 15,122) marked by their classic markers *EPCAM* and *KRT19*, natural killer (NK) cells (*n* = 3,853) identified by *GNLY* and *NKG7*, mesenchymal stromal cells (MSCs, *n* = 3,444) marked by *PDGFRB* and *COL3A1*, ECs (*n* = 2,676) characterized by expression of *PECAM1* and *VWF*, plasma cells (*n* = 2,552) positive for *IGHG1* and *MZB1*, B cells (*n* = 2,222) identified by *CD79A* and *MS4A1*, myeloid cells (*n* = 2,212) expressing *LYZ* and *FCGR3A*, cancer cells (*n* = 879) characterized by *TP63* and *KRT17* [25] and mast cells (*n* = 148) marked by *KIT* and *CPA3*. UMAP plots showing expression level of typical marker genes indicate the accuracy of our clustering. (Fig. 1c) All the 10 main cell types were present in the tumor tissues of the 8 patients. Nevertheless, depending on different subtypes and progression of the tumor of the 8 patients, the grade of infiltration of 10 main cell types varied (Fig. 1d).

### Characteristics of TAMs in the TME of human breast cancer

**Fig. 2:**
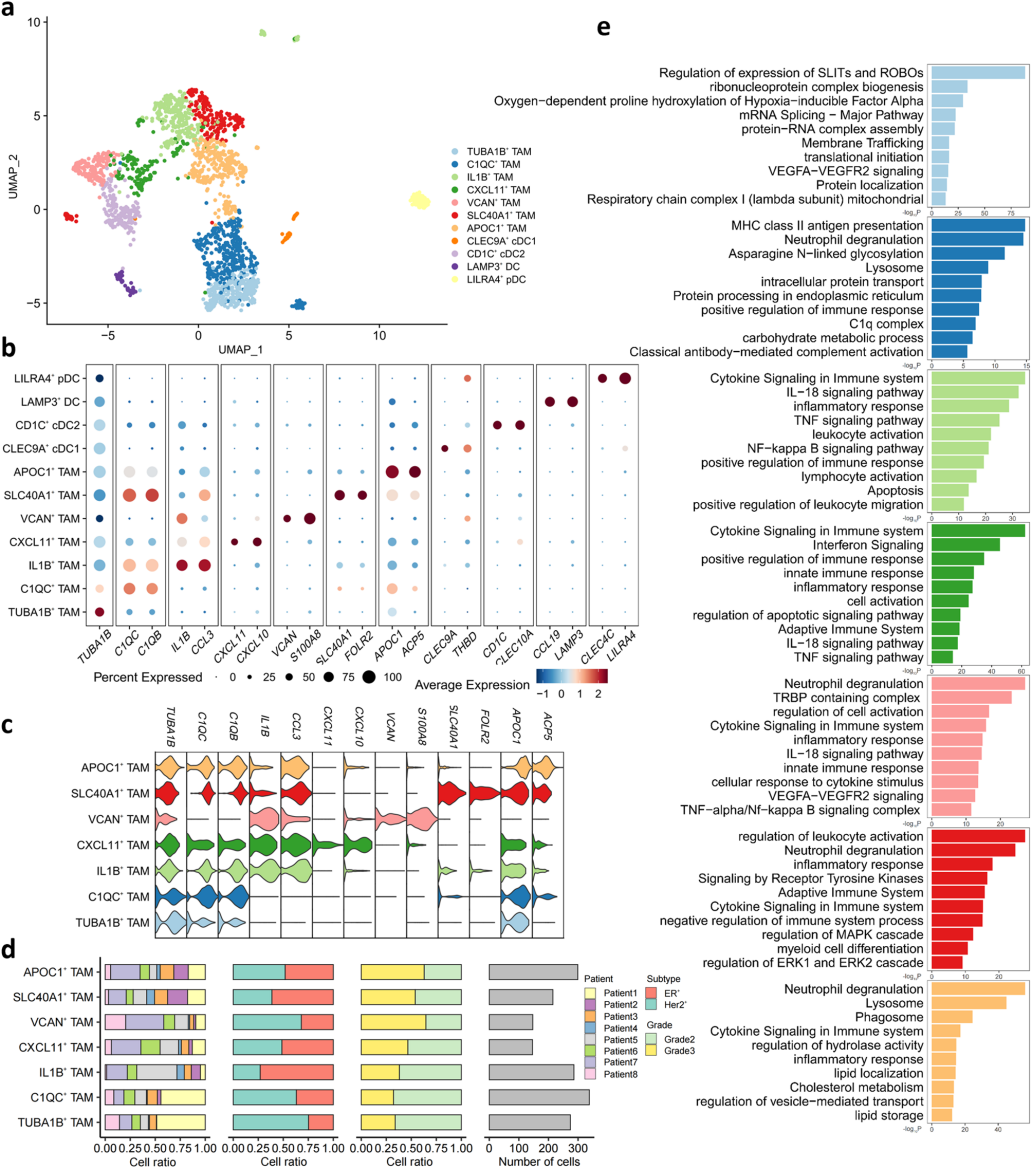
Characterization of myeloid cell subsets in human breast cancer. **a** UMAP showing the composition of myeloid cells colored by cluster. **b** Dot plot showing expression levels of selected genes in breast cancer myeloid cells. Dot size indicates fraction of cells expressing the markers, colored based on normalized expression levels. **c** Violin plot showing the expression of signature genes for 7 TAM subtypes. Colors as in **a**. **d** Proportion of 7 TAM subtypes showing in bar plots in different donors (the first panel), subtypes (the second panel), grades (the third panel) and total cell number of each cell type (the fourth panel) are shown. **e** Bar plots showing the enrichment of specific pathways in 7 TAM subtypes. Colors as in **a**

Myeloid cells, one of the most abundant immune cells in tumor, form a crucial component of the tumor immune microenvironment, playing a critical role in modulating inflammatory responses and angiogenesis [26]. In an effort to characterize subsets of myeloid cells, we next performed an unsupervised clustering of myeloid cells. Then we re-clustered all myeloid cells and identified 11 distinct groups according to their markers (Fig. 2a, b). This comprehensive classification included 7 subtypes of TAMs and 4 subtypes of dendritic cells (DCs) clusters. Different TAM subtypes had different expression patterns (Fig. 2c & Fig S2): TUBA1B^+^ TAMs (*n* = 274) marked by *TUBA1B*, C1QC ^+^ TAMs (*n* = 338) marked by *C1QC* and *C1QB*, IL1B^+^ TAMs (*n* = 286) marked by *IL1B* and *CCL3*, CXCL11^+^ TAMs (*n* = 32) marked by *CXCL11* and *CXCL10*, VCAN^+^ TAMs (*n* = 271) marked by *VCAN* and *S100A8*, SLC40A1^+^ TAMs (*n* = 579) marked by *SLC40A1* and *FOLR2*, APOC1^+^ TAMs (*n* = 779) marked by *APOC1* and *ACP5*. Similarly, the proportion of 7 TAM subtypes varied as the progression and molecular subtypes of the tumor differ (Fig. 2f). Subsequently, our research delved into the functional heterogeneity of TAMs (Fig. 2e).

TUBA1B^+^ TAMs were characterized by high expression of *TUBA1B* (Fig. 2b, c). *TUBA1B* which encodes the protein tubulin alpha 1b is a crucial microtubule isozyme. It is involved in the formation of the cytoskeleton and associated with DNA replication, spliceosome and cell cycle [27]. Our data indicates that TUBA1B^+^ TAMs exhibit effects in cell proliferation and development, inferred by the extensive enrichment of pathways related to transcription, translation and mitosis (Fig. 2e). Additionally, TUBA1B^+^ TAMs highly expressed *HMGB1* (Fig. S2), whose protein product is believed to be released by macrophages and play a role in recruiting inflammatory cells [28], thereby demonstrating the pro-inflammatory function of the TUBA1B^+^ TAMs. Furthermore, hypoxia response pathways and Slit/Robo pathway highly associated with angiogenesis, leukocyte chemotaxis and cancer metastasis [29] were significantly upregulated, indicating the pro-tumor potential of TUBA1B^+^ TAMs [30]. Hence, TUBA1B^+^ TAMs possessing proliferative and developmental potential also exhibit pro-tumor effects.

A notable characteristic of C1QC^+^ TAMs was the high expression level of the complement protein C1q encoding genes [31] *C1QC*, *C1QB*, *C1QA* and *HLA-DRB1* (Fig. 2b, c & Fig. S2). As the initiator of complement cascade and fundamental molecule of the classical complement pathway, C1q exhibits affinity for immune complexes, pentraxins and other activators within the TME. Recent evidence suggested that C1qA, C1qB, C1qC positively influence anti-tumor through antibody-mediated immune responses [32]. Classical antibody-mediated complement activation, the complement cascade and C1q complex pathways (Fig. 2e) implicate the anti-tumor role of C1QC^+^ TAMs. Furthermore, a notable upregulation in antigen processing and presentation pathways coupled with a pronounced enhancement in lysosomal pathways illustrates the antigen presentation and phagocytosis function of C1QC^+^ TAMs. To sum up, C1QC^+^ TAMs capable of presenting antigens and engulfing tumor cells exert anti-tumor effects within the tumor-immune landscape.

IL1B^+^ TAMs were characterized by the elevated expression of inflammatory factor and chemokine genes [15], such as *IL1B*, *CCL3L1*, *CCL3*, *CXCL8* and *CXCL3* (Fig. 2b & Fig. S2). CCL3L1 and CCL3, as ligands for chemokine receptors CCR1, CCR3 and CCR5 [33], play a crucial role in recruiting lymphocytes and monocytes. CXCL3 is crucial in granulocyte recruitment to injured and infected sites by interacting with CXCR3 [34]. The regulation of leukocyte migration, the activation of leukocytes and lymphocytes as well as the positive modulation of inflammatory responses (Fig. 2e) underscore the immune activity of IL1B^+^ TAMs. Anti-tumor immune pathways such as IL-18 signaling pathway [35] and TNF signaling pathway indicate its activated state of immune engagement. Consequently, IL1B^+^ TAMs actively recruit and modulate immune cells thereby orchestrating the inflammatory responses essential for eradicating tumor cells.

CXCL11^+^ TAMs highly expressed IFN regulatory genes [36] *CXCL11*, *CXCL10*, *CXCL9* and *ISG15* (Fig. 2b, c & Fig. S2). With a significant enrichment in interferon signaling (Fig. 2e), CXCL11^+^ TAMs play a regulatory role in IFN-related immune responses. Innate and adaptive immunity as well as apoptosis signaling were highly upregulated, illustrating the M1 macrophage characteristics of CXCL11^+^ TAMs [15]. Additionally, IL-18 signaling pathway and TNF pathway clarifying further their anti-tumor role. However, TAMs with high expression level of IFN genes has been previously reported to suppress anti-tumor immune responses by tryptophan degradation and recruitment of regulatory T cells (Tregs) [37]. To sum up, CXCL11^+^ TAMs may play a dual role in tumor immunity, with their impact on tumor progression under specific conditions warranting further elucidation.

A distinctive feature of VCAN^+^ TAMs was the high expression level of angiogenic features including *VCAN* and *THBS1* (Fig. 2b, c & Fig. S2), which are often upregulated in the hypoxic regions of the cancer TME [38] Apart from the upregulation of VEGFA-VEGFR2 (Fig. 2e) signaling pathway directly influencing angiogenesis, transactivation response element RNA-binding protein (TRBP) pathways are also capable of promote vascularization by TRBP binding to specific mRNA [39]. Pan-cancer analysis of TCGA dataset revealed a correlation between the abundance of TAMs expressing angiogenic genes and poor prognosis for cancer [36]. Those suggest that VCAN^+^ TAMs enhance angiogenesis, thus possibly promoting tumor growth and infiltration. Besides that, VCAN^+^ TAMs also exhibited elevated expression of the calcium-binding protein S100 family genes *S100A8* and *S100A9* (Fig. 2b, c & Fig. S2). S100 family members S100A8/A9 facilitating the migration of monocytes and neutrophils [40] are capable of inducing pro-inflammatory cytokines in monocytes and macrophages via the NF-κB and p38 MAPK pathways [41], thus promoting migration and invasion of tumor cells through Akt and p38 MAPK signaling [42]. Given the evidence, enrichment in related pathways reveals that VCAN^+^ TAMs may be associated with the chronic inflammatory environment in the TME, thus contributing to tumor progression. Therefore, VCAN^+^ TAMs exert pro-tumor roles through angiogenesis and inflammatory response.

SLC40A1^+^ TAMs exhibited high expression level of the ferroportin gene *SLC40A1* (Fig. 2b, c), which promotes the production of pro-inflammatory cytokines such as IL-6 and IL-23 while inhibiting the production of the key inflammatory regulator IL-1β in the TME, leading to the poor prognosis for cancer [43]. Signaling by receptor tyrosine kinases [44], MAPK and ERK1/ERK2 cascades regulation [45] associated with the tumor progression were significantly upregulated (Fig. 2e). In addition, *FOLR2* was also highly expressed in SLC40A1^+^ TAMs (Fig. 2b, c). Negative regulation of immune system process enriched, consistent with the fact that TAMs expressing high levels of *FOLR2* inducing the conversion to Tregs facilitate the formation of an immunosuppressive TME [46]. Notably, SLC40A1^+^ TAMs with characteristics of resident-tissue macrophages (Fig. S2) showed significant enrichment in myeloid cell differentiation (Fig. 2e), possessing lower monocyte developmental potential [36, 47]. To summarize, SLC40A1^+^ TAMs exhibit pro-tumor effects [48] as highly differentiated myeloid cells.

APOC1^+^ TAMs were characterized by the significant expression of lipid-associated genes, including *APOC1*, *ACP5* and *APOE* (Fig. 2b, c& Fig. S2). Lipid synthesis and metabolism play a crucial role in tumor development and progression. Macrophage lipid synthesis is linked to inflammation and immunity, while lipid metabolism is related to immunosuppressive and tolerance-related functions [49, 50]. APOC1^+^ TAMs exhibited notable enhancement in pathways related to lipid storage, localization and metabolism, as well as regulation of lysosome and hydrolase (Fig. 2e). TAMs with lipid-associated signature gene have been reported to promote tumor progression by taking up tumor-derived lipids through endoplasmic reticulum stress [51]. Thus, APOC1^+^ TAMs facilitate the construction of tumor tolerance environment by modulating lipid metabolism. APOC1^+^ TAMs also exhibited high expression level of *SPP1* (Fig. S2), a key gene in macrophage polarization linked to poor prognosis for cancer [52]. It is noteworthy that previous pan-cancer studies have demonstrated a ubiquitous co-expression of *SPP1* and angiogenesis features within TAMs [36], contributing to angiogenesis. Nevertheless, this phenomenon was not observed in breast cancer.

Overall, we identified 7 subtypes of TAMs in human primary breast cancer tissues. By analyzing their specific signature genes and conducting enrichment analysis, the roles these TAMs play in tumor immunity are clarified.

### TAM subtypes exhibit specificity to breast cancer molecular subtypes and progression

Our analysis of clinical information from 8 patients showed that the proportions of these 7 TAM subtypes varied among various breast cancer molecular subtypes and grades of 8 patients. Although the limited sample size prevents achieving statistical significance, we observed trends in the distribution of 7 TAM subtypes across different breast cancer molecular subtypes and progression (Fig. 2d & Fig. S3). TUBA1B^+^ TAMs, C1QC^+^ TAMs and VCAN^+^ TAMs were more prevalent in HER2^+^ breast cancer, whereas IL1B^+^ TAMs, SLC40A1^+^ TAMs and APOC1^+^ TAMs were more dominant in ER^+^ breast cancer, which illustrates the heterogeneity of TAM subtypes in various molecular subtypes of breast cancer. Additionally, we found that TUBA1B^+^ TAMs, C1QC^+^ TAMs and IL1B^+^ TAMs were predominant in early-stage tumors, whereas VCAN^+^ TAMs, SLC40A1^+^ TAMs and APOC1^+^ TAMs had higher proportions in patients with advanced cancer. This suggests that the former three TAMs play crucial roles in the early stages of tumorigenesis through expansional and developmental function of TUBA1B^+^ TAMs as well as anti-tumor effects of C1QC^+^ TAMs and IL1B^+^ TAMs. In contrast, the latter three may be significantly involved in tumor progression through their pro-tumor roles. Notably, the proportion of CXCL11^+^ TAMs did not vary significantly across different molecular subtypes and stages of breast cancer, possibly due to its dual role in tumor immunity.

### Developmental trajectory of TAMs in the TME of human breast cancer

**Fig. 3:**
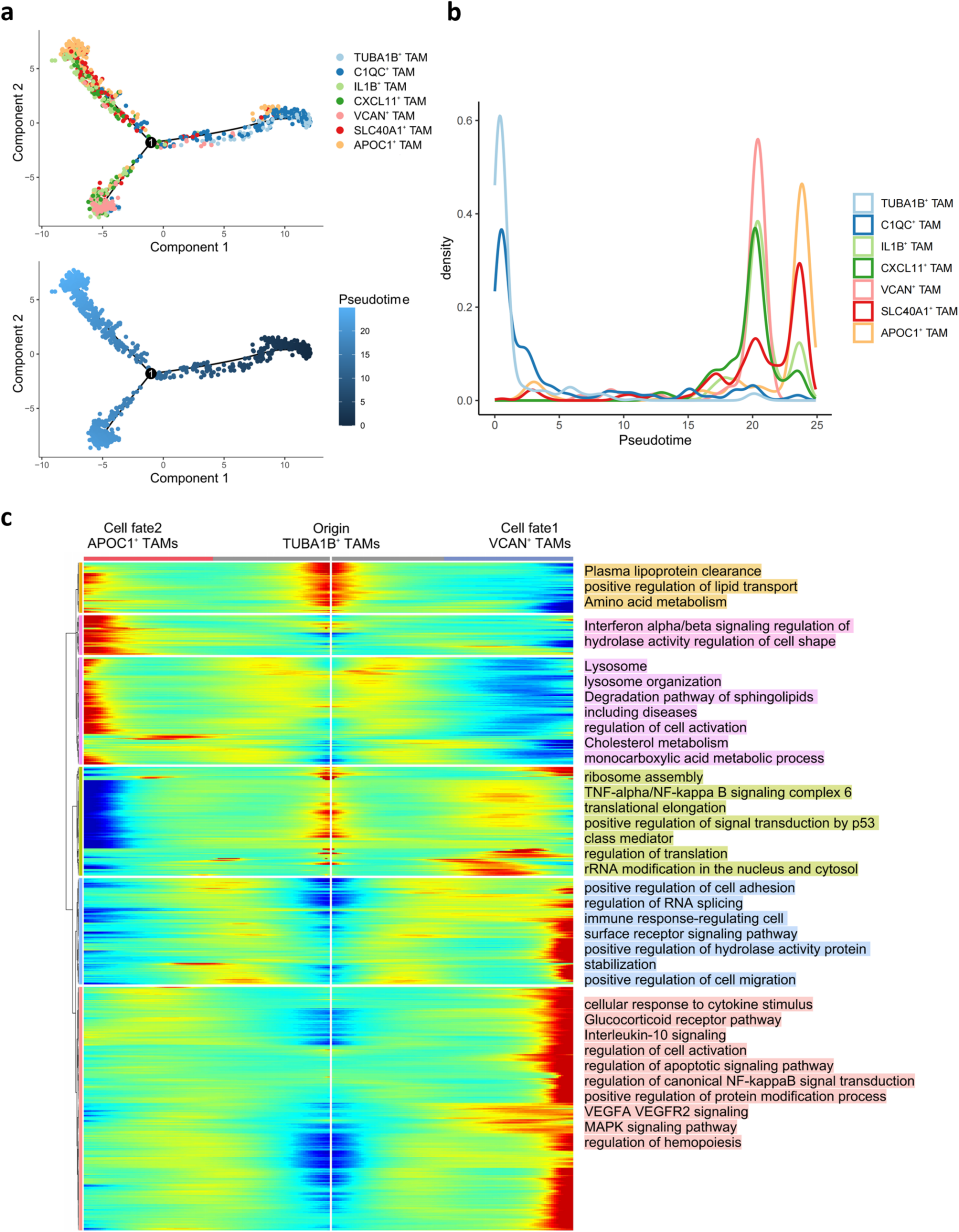
Developmental trajectories of different TAM subtypes with pseudotime analysis. **a** Pseudotime-ordered analysis of TAMs from human breast cancer samples. TAM subtypes are labeled by colors. **b** Patterns of cell density along with the pseudotime. **c** Heatmap showing the dynamic transcriptional changes along the pseudotime

Non-malignant cells are reshaped in the TME, undergoing phenotypic and functional transformations [14]. To explore how TAMs undergo transitions, we next constructed an unsupervised pseudotime trajectory based on gene signatures of all TAMs with prior clustering information. In this analysis, TUBA1B^+^ TAMs were positioned at the initiation point of the trajectory curve (Fig. 3a). TUBA1B^+^ TAMs with high expression of cytoskeleton genes demonstrate its expansional and developmental potential (Fig. 2b, e). Hence, TUBA1B^+^ TAMs are inferred to serve as a potential origin for other TAMs. The trajectory branched into two distinct cell fates along with psudotime, culminating in VCAN^+^ TAMs and APOC1^+^ TAMs.

It is noteworthy that the cell densities of TAMs also varied along the developmental trajectory (Fig. 3b & Fig. S4). TUBA1B^+^ TAMs initially dominated and subsequently declined. Meanwhile, cell densities of other TAMs increased, indicating TUBA1B^+^ TAMs might transform into other TAMs. Moreover, C1QC1^+^ TAMs exhibiting anti-tumor roles played a crucial role in the early stage of the psudotime. The cell densities of IL1B^+^ TAMs, CXCL11^+^ TAMs and VCAN^+^ TAMs gradually increased along with pseudotime. Towards the end of the pseudotime, SLC40A1^+^ TAMs and APOC1^+^ TAMs functioning pro-tumor effects began to prevail. This observation suggests that within the breast cancer TME, the dominant TAM subtype shifts from anti-tumor phenotypes to pro-tumor phenotypes along with the psudotime.

To understand transcriptional changes associated with transitional states, we next explored how gene expression shifts from the initial state to two distinct cell fates (Fig. 3c). Unsupervised clustering categorized DEGs into six clusters, each representing a unique transformation pattern. The transcriptional changes from the starting state to two different cell fates were characterized by three gene sets corresponding to each trajectory. The transition of TUBA1B^+^ TAMs to VCAN^+^ TAMs involved three modules - cell activation and angiogenesis, translational processes and signal transduction by p53 class mediator, as well as cell adhesion and cell surface receptor signaling pathways. Conversely, the transition of TUBA1B^+^ TAMs to APOC1^+^ TAMs was characterized by three distinct modules - regulation of hydrolase activity and cell shape alteration, cell activation and lipid metabolism, as well as lipoprotein metabolism and lipid localization. Collectively, these gene sets provide biological insight into the morphological and functional transformations of transition from TUBA1B^+^ TAMs to two cell fates.

### Cell-cell communication networks of TAMs and other cells in the TME

We next utilized the cellphoneDB to study the ligand-receptor specificity between TAMs and other cell clusters, investigating their interactions within the TME. Several signals including TNF, IFNs, NF-κB signaling pathway, hypoxic conditions are capable of modulating the activation, phenotypic and functional transformation of macrophages [53]. TAMs not only undergo metabolic adaptative changes, but also influence the functional state of other cells within the TME [54]. By analyzing the interaction between TAMs and all other cells within the TME, we found that ECs and T cells had the most significant protein pairs with TAMs (Fig. S5), demonstrating pronounced interaction.

### Characteristics of ECs and cell-cell interaction with TAMs

**Fig. 4:**
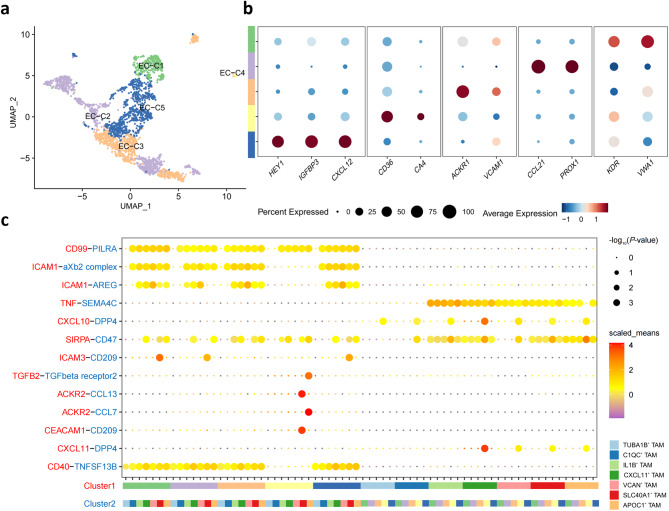
Characterization of EC subsets and cell-cell interaction with TAMs. **a** UMAP showing the composition of ECs colored by cluster. **b** Dot plots showing expression levels of selected genes in breast cancer ECs. Dot size indicates fraction of cells expressing the markers, colored based on normalized expression levels. Colors as in **a**. **c** Overview of selected ligand-receptor interactions of ECs and 7 types of TAMs. *P*-values are indicated by circle size, with the scale to the right. The means of the average expression levels of interacting molecule in cluster 1 and interacting molecule in cluster 2 are indicated by color. Assays were carried out at the mRNA level but were used to extrapolate protein interactions. Colors as in **a**

Unsupervised clustering of ECs identified 14 groups, which we merged into five EC subtypes (Fig. 4a) based on marker genes of traditional vascular bed EC subtypes reported previously [55]. Tumor vasculature plays a crucial role in the growth, invasion and metastasis of tumors [56]. The vascular heterogeneity in the progression of breast cancer is closely associated with angiogenesis, arterial, capillary, venous and lymphatic EC phenotypes [57]. EC-C1 exhibited high expression of arterial characteristics (*HEY1*, *IGFBP3*, *CXCL12*), EC-C2 showed capillary features (*CD36*, *CA4*), EC-C3 exhibited venous characteristics (*ACKR1*, *VCAM1*), EC-C4 expressed lymphatic features (*CCL21*, *PROX1*) and EC-C5 exhibited angiogenic traits (*KDR*, *VWA1*) (Fig. 4b). We then speculated on extensive interactions between pro-tumor TAM subtypes and ECs through significantly upregulated ligand-receptor pairs.

ICAM1 is regarded as a principal regulator of inflammatory responses and tumorigenesis, playing a critical role in inducing angiogenesis [58]. The significant upregulation of ICAM1-AREG elucidates the promotive effects of VCAN^+^ TAMs on angiogenesis and tumor development (Fig. 4c). As a carcinoembryonic antigen (CEA) and a member of the immunoglobulin superfamily, CEACAM1 plays an essential role in the development of ECs, crucial for the formation of tumor vasculature and lymphatics [59]. CEACAM1-CD209 reveals the propelling role of SLC40A1^+^ TAMs in tumor angiogenesis and lymphangiogenesis. Epithelial-mesenchymal transition (EMT) is closely associated with the invasiveness and distant metastasis of cancer cells [60], which can be induced by TGFβ2 via the TGFβ/SMAD signaling pathway [61]. A similar process, endothelial-mesenchymal transition (EndMT), is also a vital mechanism in angiogenesis. Previous reports have highlighted the significant role of the TGF-β pathway in promoting angiogenesis and progression in breast cancer by inducing EndMT [62], thereby clarifying the promotive effect of the TGFB2-TGFBR2 on APOC1^+^ TAMs in tumor progression. TAMs possess the ability to regulate the phenotype and function of ECs. Conversely, ECs in turn are capable of modulating the recruitment and the polarization of TAMs [63]. The CD47-SIRPα pathway is a crucial immune checkpoint in tumor progression. Once activated, it will cause immunosuppression in macrophages and inhibits the ability of macrophages and DCs to kill tumor cells [64]. The significant upregulation of SIRPA-CD47 confirms the regulatory effect of ECs on TAMs and also reveals the immunosuppressive action of APOC1^+^ TAMs against anti-tumor immunity. Lymphatic ECs with high *ACKR2* expression have the capacity to recruit inflammatory cells effectively [65]. The interaction between ACKR2 and CCL13, CCL7 illustrates that SLC40A1^+^ TAMs and APOC1^+^ TAMs be recruited by ECs to modulate tumor immunity. Furthermore, the immunosuppressive effect of CXCL11^+^ TAMs is revealed. Previous studies have reported the negative impact of DPP4 on anti-tumor immunity, where inhibiting DPP4 can enhance the efficacy of tumor immunotherapy [66]. EC-C4 highly expressing DPP4 interacted with TAMs expressing chemokines CXCL10 and CXCL11, especially CXCL11^+^ TAMs, thereby inhibiting their anti-tumor immune response.

### Characteristics of T cell and cell-cell interaction with TAMs

**Fig. 5:**
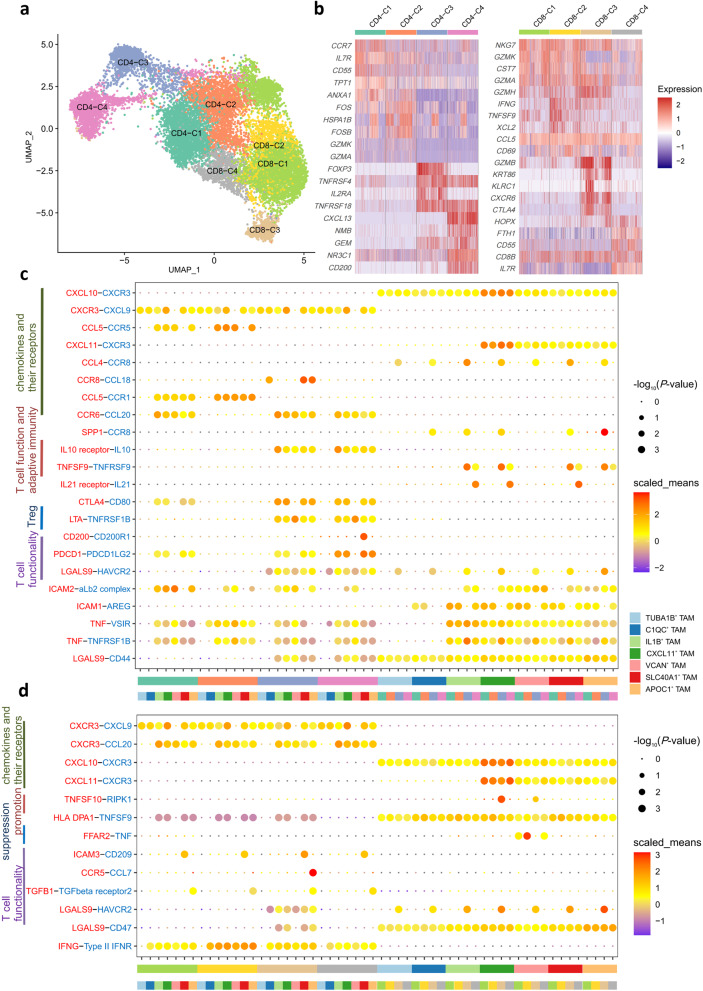
Characterization of T cell subsets and cell-cell interaction with TAMs. **a** UMAP showing the composition of T cells colored by cluster. **b** Heatmap showing genes that are differentially expressed across 8 T cell subtypes. Colored based on normalized expression levels. **c** and **d** Overview of selected ligand–receptor interactions of CD4^+^ T cells (**c**), CD8^+^ T cells (**d**) and 7 types of TAMs. *P*-values are indicated by circle size, with the scale to the right. Colors as in **a**

T cells exert a crucial function within the TME, capable of infiltrating the TME and recognizing cancer cell epitopes [67]. Unsupervised clustering analysis of T cells categorized them into 8 distinct clusters, which we merged into 4 CD4^+^ T cell subtypes and 4 CD8^+^ T cell subtypes based on antigen surface markers and specific genes (Fig. 5a). CD4-C1 characterized by high expression of both *CCR7* and the anti-inflammatory marker *ANXA1* were defined as central memory T cells [68] (Fig. 5b). CD4-C2 expressed elevated levels of FOS family genes *FOS* and *FOSB* as well as cytotoxic markers *GZMK* and *GZMA*. CD4-C3 with high expression of *FOXP3* [69] were categorized as Tregs. CD4-C4 distinguished by high *CXCL13* expression level were identified as CXCL13^+^ follicular helper T cells, which have been reported to regulate B cell chemotaxis and play a critical role in transitioning from Treg-mediated immune suppression to adaptive anti-tumor humoral responses [70]. CD8-C1 marked by the expression of cytotoxic markers *NKG7*, *GZMA*, *GZMK* and *GZMH* were categorized as effector memory T cells [71]. CD8-C2 characterized by high *CD69* expression were identified as CD69^+^ tissue-resident memory T cells [72]. CD8-C3 expressing both cytotoxic markers *GZMB* and exhaustion marker *CTLA4* [73] were defined as exhausted CD8^+^ T cells. Lastly, CD8-C4 with a distinct expression profile of cytotoxic markers and *IL7R* was identified as IL7R^+^ memory T cells [68]. Previous studies have demonstrated that TAMs within the TME can interact with CD4^+^ T cells and notably suppress the activation of NK cells and CD8^+^ T cells [54]. To further explore these dynamics, we identified critical ligand-receptor pairs and molecular interactions between TAMs and T cells.

Interaction between TAMs and CD4^+^ T cells were categorized into 4 groups - the role of chemokines and their receptors in infiltration, modulation of T cell function and adaptive immunity, regulation of Tregs for immune evasion and impact on T cell functionality. TAMs universally expressed chemokines including CCL20, CXCL9 and CXCL16, while corresponding chemokine receptors were highly expressed in CD4^+^ T cells (Fig. 5c). It indicates TAMs extensively facilitate the infiltration of CD4^+^ T cells. The interaction between IL1B^+^ TAMs and CD4^+^ T cells was notably upregulated via the CCL20-CCR6 axis related to inflammation [74], which illustrates the inflammatory functions of IL1B^+^ TAMs. Furthermore, the IL10 receptor-IL10 pair emerged as a critical receptor-ligand mediating the interaction between IL1B^+^ TAMs and CD4^+^ T cells. The anti-tumor effect of IL-10 has been demonstrated in various tumor models through the activation of T cells [75], partially demonstrating the anti-tumor role of IL1B^+^ TAMs. Mouse tumor models have proven that TNFRSF9 enhances anti-tumor immune responses by strengthening the functionality of CD4^+^ T cell, thereby amplifying the effectiveness of CD8^+^ T cell-mediated responses [76]. Thus, IL1B^+^ TAMs and CXCL11^+^ TAMs are proven to interact with CD4-C3 through TNFSF9 − TNFRSF9, thereby enhancing adaptive immune responses. TNFRSF1B, a member of the TNF receptor superfamily highly expressed in Tregs, possesses specificity and serves as a potential driver of immune evasion and tumor proliferation [77]. The significant upregulation of LTA − TNFRSF1B between CD4-C3 and VCAN^+^ TAMs suggests that VCAN^+^ TAMs may facilitate tumor immune suppression and proliferation through interactions with Tregs. Moreover, interaction between inhibitory co-stimulatory molecule PDCD1 and PDCD1LG2 reveal the suppressive effect on immunity of SLC40A1^+^ TAMs and APOC1^+^ TAMs. CD200-CD200R1 contributing to tumor growth and progression [78] indicates the pro-tumor function of SLC40A1^+^ TAMs by interacting with CD4-C4. In addition, hepatitis A virus cell receptor 2 gene *HAVCR2* encodes the transmembrane receptor TIM-3, whose expression is significantly associated with T cell dysfunction and exhaustion [79]. The interaction between TIM-3 and galactoglucan lectin 9 (LGALS9) has been shown to induce apoptosis in Th1 cells, thereby reducing anti-tumor immune responses [80]. Therefore, APOC1^+^ TAMs and CXCL11^+^ TAMs potentially suppress anti-tumor immunity by inducing T cell dysfunction, exhaustion or even apoptosis through LGALS9 − HAVCR2 pair.

Similar to the situation in TAMs and CD4^+^ T cells, cell-cell communication between TAMs and CD8^+^ T cells were also divided into 4 categories - the role of chemokines and their receptors in infiltration, promotion of anti-tumor immunity, suppression of anti-tumor immunity and impact on T cell functionality. Chemokines such as CCL3, CXCL11, CXCL10 and CXCL9 along with their receptors CXCR3 and CCR5 were also widely distributed in TAMs and CD8^+^ T cells (Fig. 5d), indicating that TAMs generally influence the infiltration of CD4^+^ T cells. TNFSF10 (TRAIL) whose coding gene is a target gene transcribed by p53 mediates p53-dependent cell death [81]. Previous mouse models have shown that activation of TRAIL can lead to the activation of RIPK1, thereby inducing inflammatory responses [82] and driving cell death [83], explaining the anti-tumor role of CXCL11^+^ TAMs by eliminating tumor cells through TNFSF10-RIPK1. Ligands for FFAR2 (G protein-coupled receptor 43, GPR43) inhibit the TNF-α signaling pathway [84], thus FFAR2 − TNF being upregulated signifies the suppressive effect of VCAN^+^ TAMs on anti-tumor immunity. CD209 (DC-SIGN), a receptor for ICAM-3, mediates transient adhesion with T cells, thereby suppressing T cell function [85], indicating the immunosuppressive role of SLC40A1^+^ TAMs. High expression of CCL7, a natural antagonist for CCR5 [86], demonstrates the role of APOC1^+^ TAMs in inhibiting the mobility of CD8^+^ T cells, thus suppressing tumor immune responses. Besides, once TGFβ receptor 2 binds to TGFβ1, TGF-β signaling pathway will be activated [87], affecting the functioning of CD8^+^ T cells and leading to suppression of T cell immunity [88]. Furthermore, the binding of TIM-3 to its ligand Galectin-9 not only impacts Th cells, but is also associated with CD8^+^ T cell exhaustion [89], further illustrating the immunosuppressive function of APOC1^+^ TAMs by inhibiting T cell function and inducing T cell exhaustion.

### Impact of TAMs on the prognosis of tumors revealed by clinical data

**Fig. 6:**
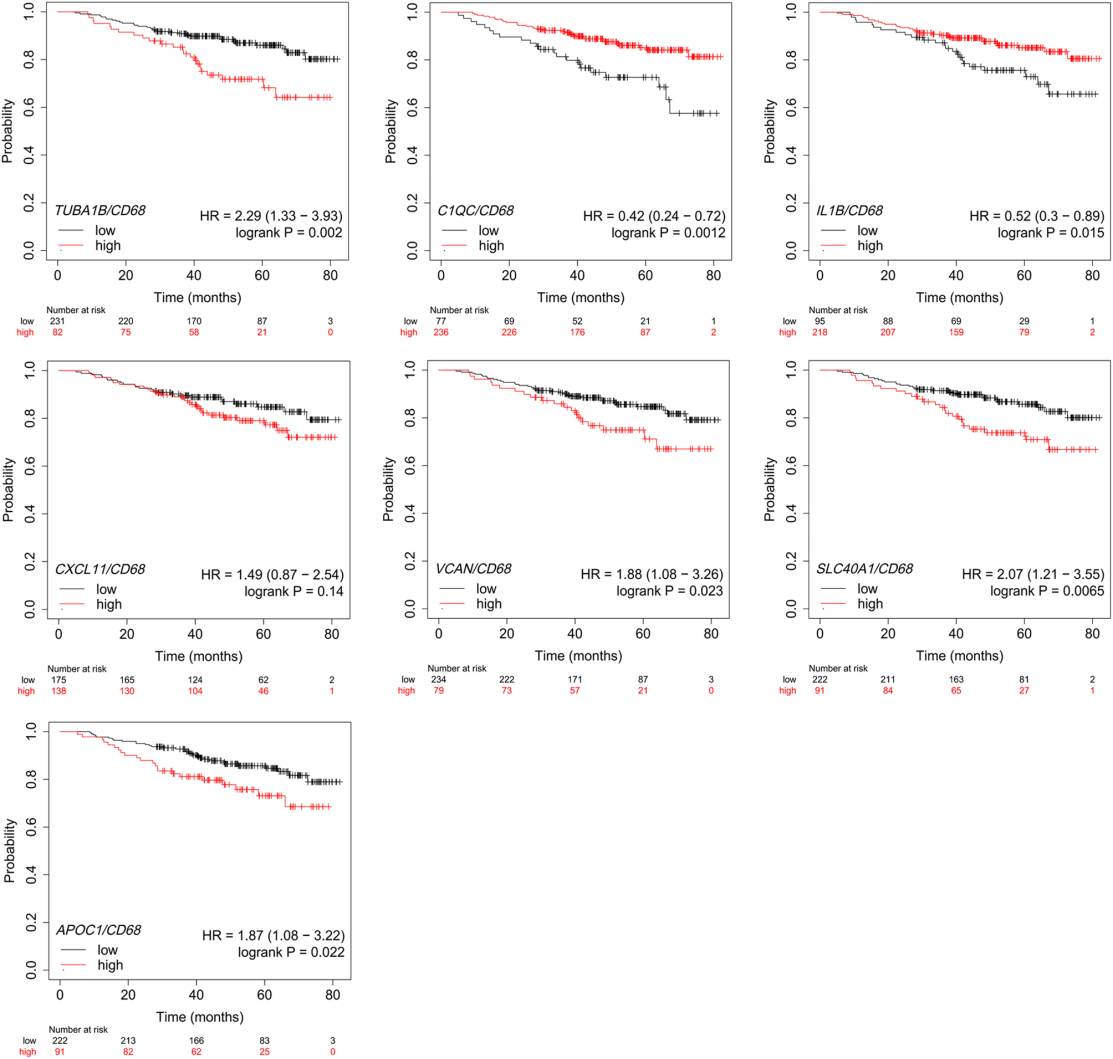
Correlations of TAM subtype-specific genes with OS in breast cancer. Kaplan–Meier survival curves for OS in 313 breast cancer patients who had not undergone endocrine therapy or chemotherapy from TCGA BRCA. Stratification based on high vs. low expression of 7 TAM signature genes (*TUBA1B*, *C1QC*, *IL1B*, *CXCL11*, *VCAN*,* SLC40A1* and *APOC1*). Log rank test

The clinical data from 8 patients indicates that TUBA1B^+^ TAMs, C1QC^+^ TAMs and IL1B^+^ TAMs play a significant role in early-stage tumors, whereas VCAN^+^ TAMs, SLC40A1^+^ TAMs and APOC1^+^ TAMs dominate in advanced tumors (Fig. 2d). To investigate the clinical relevance of TAMs, we conducted a survival analysis using the Cancer Genome Atlas (TCGA) Breast Cancer (BRCA) dataset. Patients who had not received endocrine therapy or chemotherapy were included in this survival analysis. Since *C1QC*, *IL1B* and *CXCL11* were virtually absent in other cell types (Fig. S6), these signature genes are capable of accurately reflecting the abundance of TAMs. To further characterize the prognostic impact of the other four TAM subtypes, we conducted a survival analysis on the signature gene sets (Fig. S2) associated with these TAMs (Fig. S7). Patients with high level of C1QC^+^ TAMs and IL1B^+^ TAMs were associated with better OS, whereas those with high level of TUBA1B^+^ TAMs, CXCL11^+^ TAMs, VCAN^+^ TAMs, SLC40A1^+^ TAMs and APOC1^+^ TAMs were associated with worse OS (Fig. 6 and Fig. S7).

### Characteristics of TAMs validated by other publicly available independent scRNA datasets

**Fig. 7:**
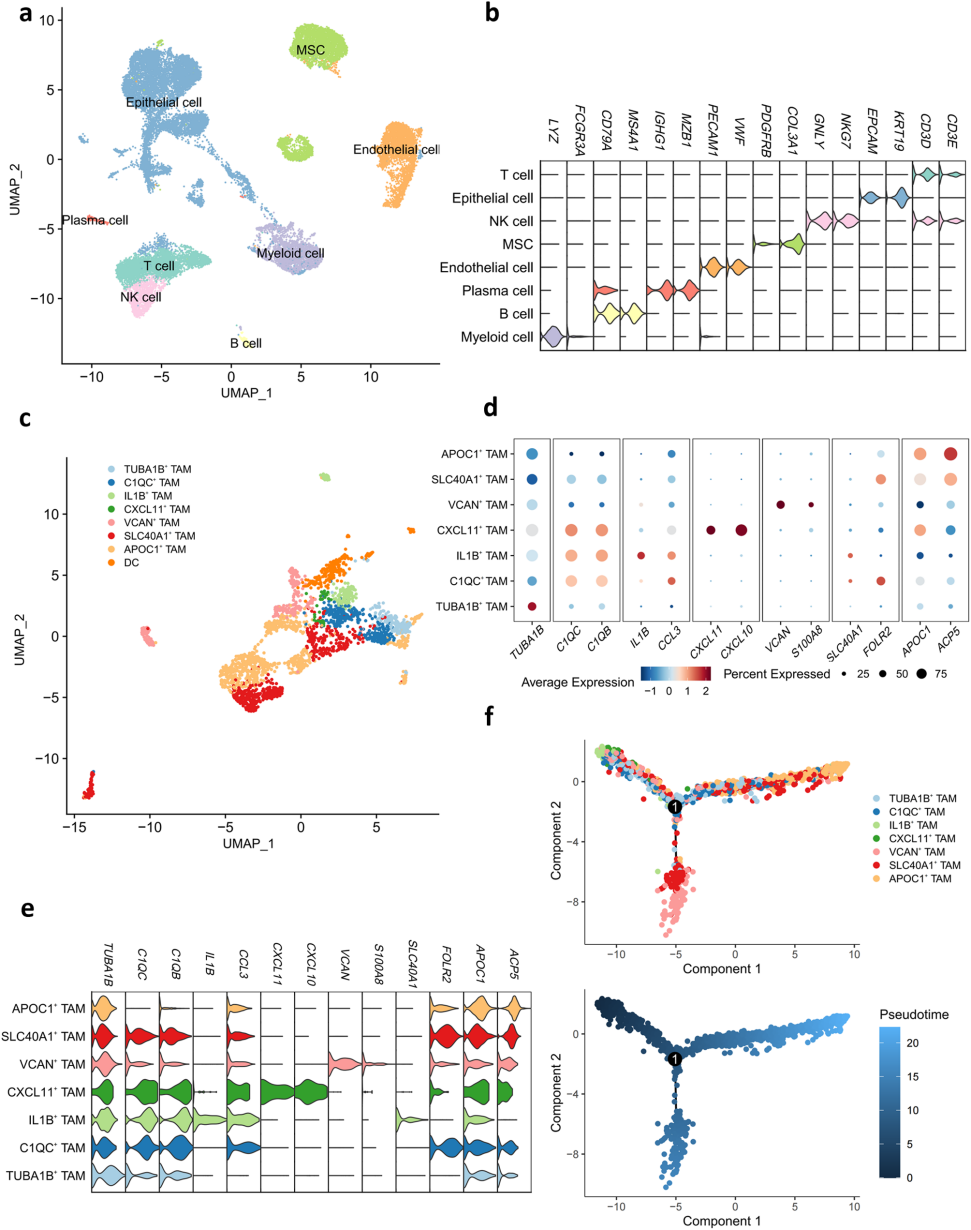
Characteristics of TAMs validated by other publicly available independent scRNA datasets. **a** UMAP plot of 25,925 cells from tumor tissue of the 4 untreated breast cancer patients, showing 8 clusters in the plot. Each cluster was shown in different color. **b** Violin plot showing the expression of selected signature genes of main cell types. Colors as in **a**. **c** UMAP showing the composition of myeloid cells colored by cluster. **d** (Dot plot) and **e** (Violin plot) showing expression levels of selected genes in 7 TAM subtypes. **f** Pseudotime-ordered analysis of TAMs from human breast cancer samples

To validate the characterization of TAMs, previous analyses were conducted on another publicly available independent scRNA-seq dataset from 4 untreated primary breast cancer tissues. By clustering 25,925 single cells, we identified 8 main cell types using previously established signature genes (Fig. 7a, b): T cells (*n* = 3,801), epithelial cells (*n* = 10,498), NK cells (*n* = 1,014) MSCs (*n* = 4,098), ECs (*n* = 3,426), plasma cells (*n* = 225), B cells (*n* = 293) and myeloid cells (*n* = 2,570). The proportions of these cells varied from those in the previous dataset due to differences in sampling. Following the initial analysis, the process of re-clustering also revealed 7 TAM subtypes with distinct expression patterns in the new dataset (Fig. 7c-e): TUBA1B^+^ TAMs (*n* = 166), C1QC^+^ TAMs (*n* = 323), IL1B^+^ TAMs (*n* = 177), CXCL11^+^ TAMs (*n* = 32), VCAN^+^ TAMs (*n* = 271), SLC40A1^+^ TAMs (*n* = 579) and APOC1 + TAMs (*n* = 779). Subsequently, developmental trajectory analysis for these 7 TAM subtypes was independently performed. Consistent with our earlier findings, TUBA1B^+^ TAMs were positioned at the initial branch of the developmental trajectory, branching into two cell fate endpoints-VCAN^+^ TAMs and APOC1^+^ TAMs (Fig. 7f), corroborating our previous conclusions (Fig. 3c).

## Discussion

scRNA-seq has emerged as a formidable tool for exploring the heterogeneity of the TME in various cancers [90]. The complexity of breast cancer has been extensively explored, leading to a progressive revelation of its TME at a single-cell level [18]. Despite these advancements, there is still a notable gap in the systematic classification and functional analysis of TAMs within the breast cancer.

**Fig. 8:**
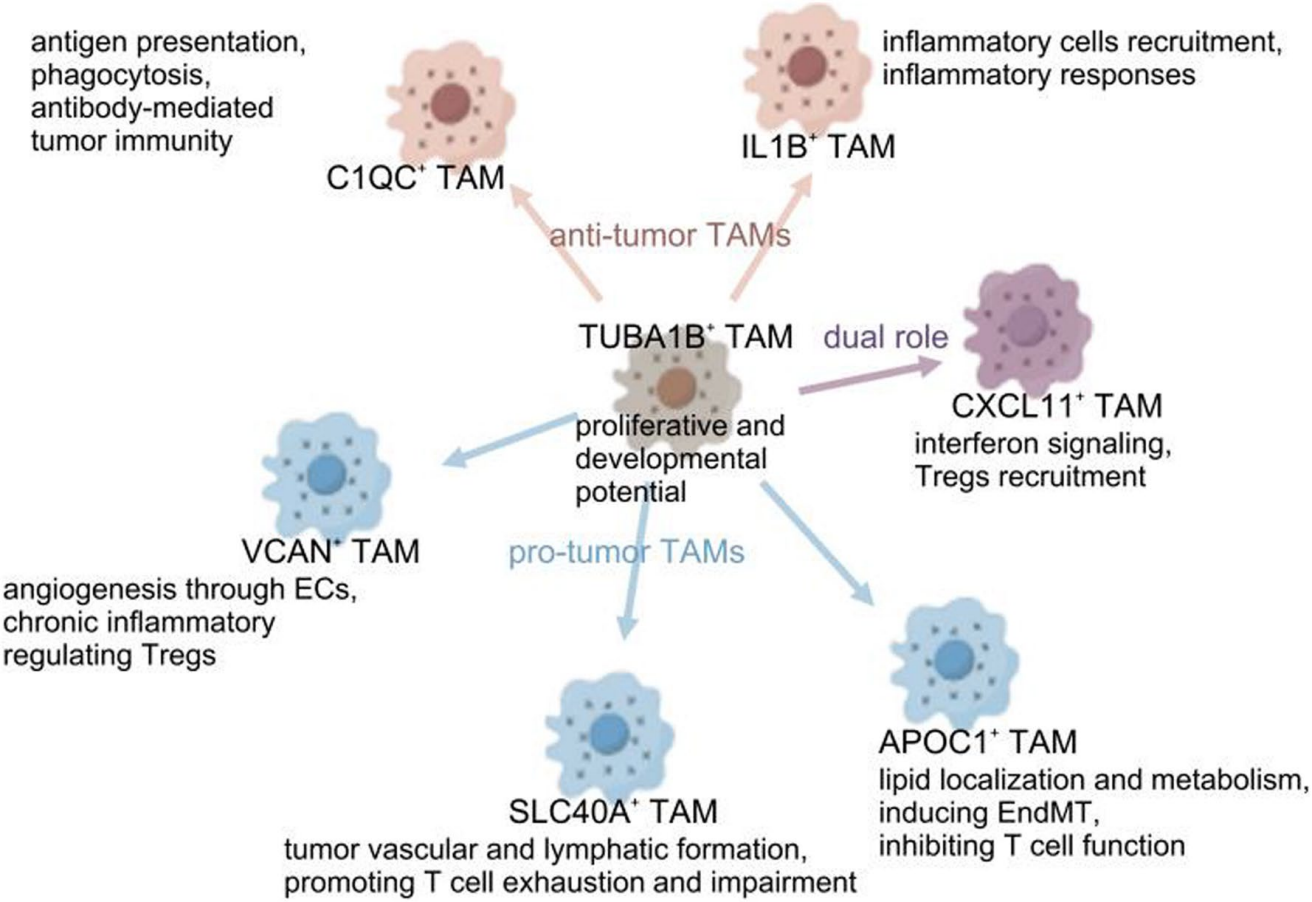
Schematic illustration of cellular interactions and functions of TAMs in the TME. TUBA1B^+^ TAMs with the proliferative and developmental ability contribute to the diversity of TAMs. They are capable of transforming into other TAM subtypes, which exert anti-tumor or pro-tumor effects through various mechanisms

In this study, we made significant strides in establishing a cell classification model for breast cancer TAMs. Analyzing public scRNA-seq data from 8 untreated primary breast cancer patients, we categorized TAMs into 7 distinct subtypes through unsupervised clustering based on specific marker genes, conducted enrichment analysis based on their DEGs, summarized the functional properties of the TAM subtypes and thus predicted the anti-tumor or pro-tumor effect they exert (Fig. 8). A key finding is the identification of a TAM subtype with proliferative and developmental ability, characterized by the expression of *TUBA1B* encoding a protein called Tubulin alpha 1b involved in cytoskeletal formation. Developmental trajectory analysis pinpoints TUBA1B^+^ TAMs as a starting point. Along with the psudotime, it transitioned into VCAN^+^ TAMs and APOC1^+^ TAMs, undergoing morphological and functional changes. Notably, although proliferative TAMs have been identified by proliferation indicator Ki-67 (*MKI67*) and cell cycle genes such as *CDK*1 [15] in various cancer models [12], such TAMs were not previously identified in breast cancer due to various gene expression across cancer types. TAMs play key roles in immune suppression, angiogenesis, ECM remodeling and tumor growth. They exert these effects not only by directly modulating innate immunity and indirectly regulating adaptive immunity, but also by interacting with other immune cells within the TME [91]. To further understand the role of different TAM subtypes in the cell-cell communication network of the TME, we analyzed the functional interplay between 7 TAM subtypes and other cell types to identify key ligand-receptor pairs and major subtypes influencing the critical functions of TAMs. We observed that TAMs universally expressed chemokines. Chemokines with their receptor were significantly upregulated in the communication network between TAMs and T cells, demonstrating that TAMs generally influence the infiltration of T cells. C1QC^+^ TAMs expressing classical complement molecules C1q genes exert anti-tumor effects through antigen presentation, phagocytosis and enhancing antibody-mediated tumor immunity. IL1B^+^ TAMs expressing inflammatory factors and chemokines promote anti-tumor immunity not only by recruiting inflammatory cells and activate inflammatory responses but also by enhancing adaptive immune responses through its interaction with T cells. Notably, CXCL11^+^ TAMs with high expression level of interferon genes play a dual role in tumor immunity. On one hand, they initiate innate immune responses and interferon signaling as well as enhance adaptive immune responses; on the other hand, they suppress T cell function, induce T cell exhaustion and exert pro-tumor roles through key immunosuppressive pathways. The remaining three subtypes contribute to immune suppression and tumor progression through distinct mechanisms. VCAN^+^ TAMs expressing angiogenic factors and calcium-binding proteins modulate chronic inflammatory responses, interact with ECs to promote angiogenesis and regulate Tregs. SLC40A1^+^ TAMs encoding iron transport proteins regulate chronic inflammation, facilitate tumor vascular and lymphatic formation through ECs and promote T cell exhaustion and impairment. APOC1^+^ TAMs expressing lipid-related genes regulate lipid localization and metabolism, induce EndMT and inhibit T cell function. Developmental trajectory revealed that TUBA1B^+^ TAMs possess potential to transform into other TAM subtypes. Transcriptomic data and clinical information corroborate the multifaceted role of TAMs in the breast cancer TME. At last, validation was conducted in other datasets, confirming the findings.

It is crucial to acknowledge the limitations inherent in our study. The data was only from a static snapshot of gene expression profile. Besides, the analysis of developmental trajectory was based on inferences drawn from transcriptomic gene expression profiles. It is unable to confirm the biological significance of the cellular origins of TUBA1B^+^ TAMs. Future technologies enabling real-time cell monitoring will enhance our understanding of the dynamic morphological and functional changes during cell development. Furthermore, it is essential to validate the cell-cell communication identified in our findings in vivo.

Our analysis of intercellular interactions was based on the inference of protein interactions from the detected mRNA. However, the actual distance between cells might be substantial in the TME. Understanding the anatomical tissue structure is crucial for comprehending these interactions. Spatial characterization of the immunological architecture within tumors will provide a more in-depth understanding of the interactions between TAMs and other cells.

## Conclusion

Our study identified 7 distinct subtypes of TAMs in the breast cancer TME using specific marker genes. Through enrichment analysis and the communication networks with other cells in the TME, we clarified the biological functions of these TAMs, revealing their potential roles in tumor suppression or promotion. Additionally, the clinical relevance of TAMs was analyzed through OS in the TCGA BRCA dataset, yielding promising results. The results of our experiment were repeated in another independent public dataset, validating our findings. Our findings offer a valuable resource for further research to gain deeper biological insights and pave the way for developing novel immunotherapeutic strategies targeting TAMs in breast cancer.

## Electronic supplementary material

Below is the link to the electronic supplementary material.

Supplementary Material 1: Figure S1 UMAP showing unsupervised clustering results of all cells from tumor tissue of breast cancer patients

Supplementary Material 2: Figure S2 Heatmap showing genes that are differentially expressed across 7 TAM subtypes

Supplementary Material 3: Figure S3 Bar plots showing percentages of 7 TAMs to total TAMs in breast cancers of different grades (A) and subtypes (B)

Supplementary Material 4: Figure S4 The patterns of cell densities of 8 patients along with the pseudotime

Supplementary Material 5: Figure S5 Cell-cell interaction between TAMs and other cells in the TME. (A) Intercellular communication capacity between TAM and other cells in the TME. The color of each line indicates the ligand expressed by the cell population of the same color. Lines are linked to cell types that express homologous receptors. The thickness of the line is proportional to the number of ligands when homologous receptors are present in the receptor cell type. Loop lines indicate autocrine circuits. The figure quantifies potential communication but does not illustrate the anatomical location or boundaries of the cell types. (B) Details of ligands expressed by each major cell type and cells expressing homologous receptors to receive signals. Numbers indicate the number of ligand-receptor pairs connected between each cell

Supplementary Material 6: Figure S6 Dot plot (A) and violin plot (B) showing expression levels of TAM subtype-specific genes (*CD68*, *TUBA1B*, *C1QC*, *IL1B*, *CXCL11*, *VCAN*, *SLC40A1* and *APOC1*) across different main cell types in breast cancer

Supplementary Material 7: Figure S7 Correlations of 4 subtypes of TAMs signature gene sets with OS. *TUBA1B*, *HMGB1*, *TMSB10*, *TMSB4X* for TUBA1B+ TAMs, *CXCL11*, *CXCL10*, *CXCL9*, *ISG15* for CXCL11+ TAMs, *VCAN*, *THBS1*, *S100A8*, *S100A9*, *FCN1* for VCAN+ TAMs and *SLC40A1*, *FOLR2*, *CD163* for SLC40A1+ TAMs

## Data Availability

The scRNA-seq dataset (GSE167036 and GSE248288) was downloaded from the GEO database (https://www.ncbi.nlm.nih.gov/geo/).
