# Supplementary figures and images for: Single-cell transcriptional atlas of tumor-associated macrophages in breast cancer
Source: Breast Cancer Res. 2024 Sep 4;26:129. doi: 10.1186/s13058-024-01887-6 (PMC11373130; doi:10.1186/s13058-024-01887-6)

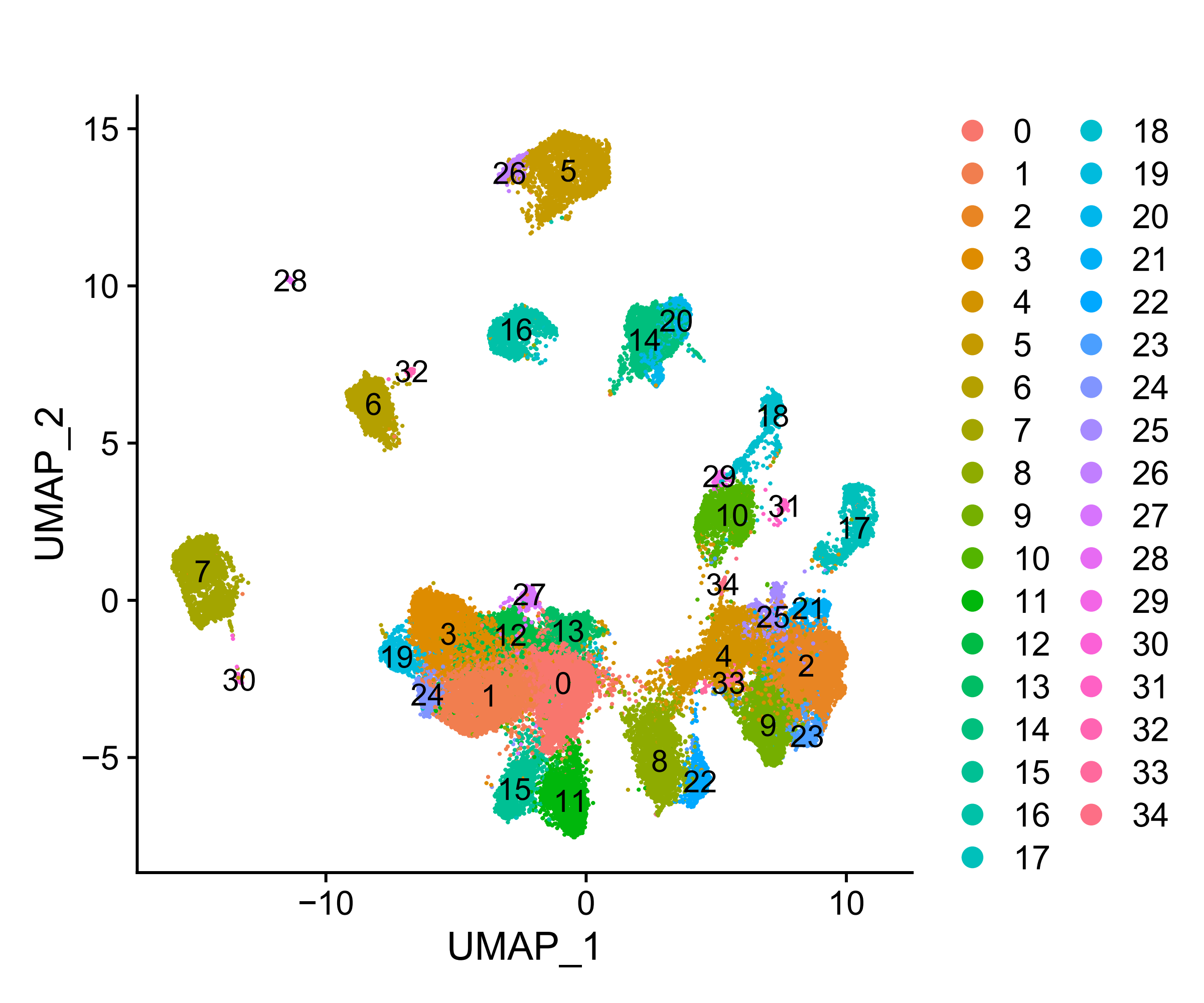

Supplement: Supplementary file 1 — Supplementary Material 1: Figure S1 UMAP showing unsupervised clustering results of all cells from tumor tissue of breast cancer patients [file 13058_2024_1887_MOESM1_ESM.tif]

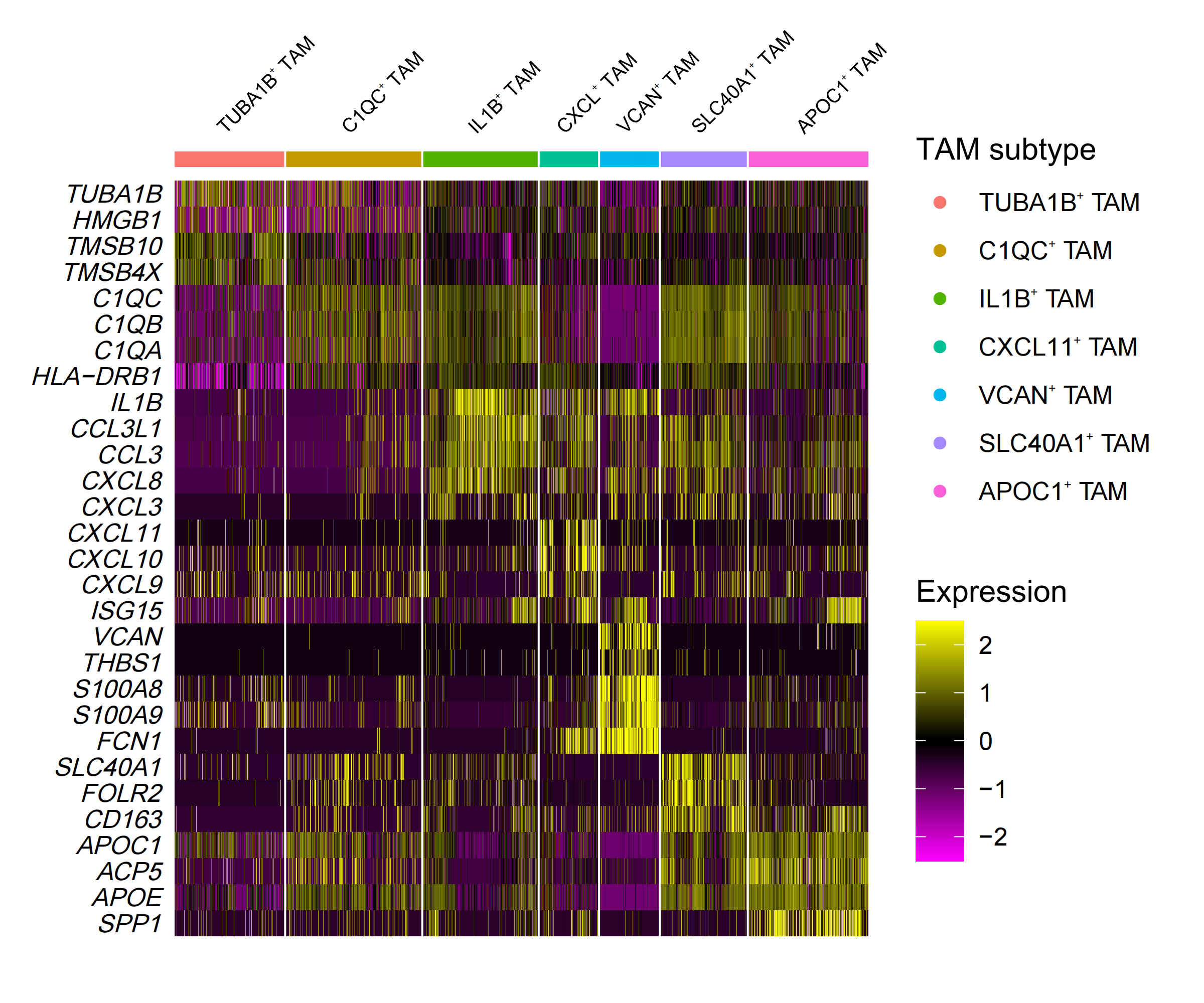

Supplement: Supplementary file 2 — Supplementary Material 2: Figure S2 Heatmap showing genes that are differentially expressed across 7 TAM subtypes [file 13058_2024_1887_MOESM2_ESM.tif]

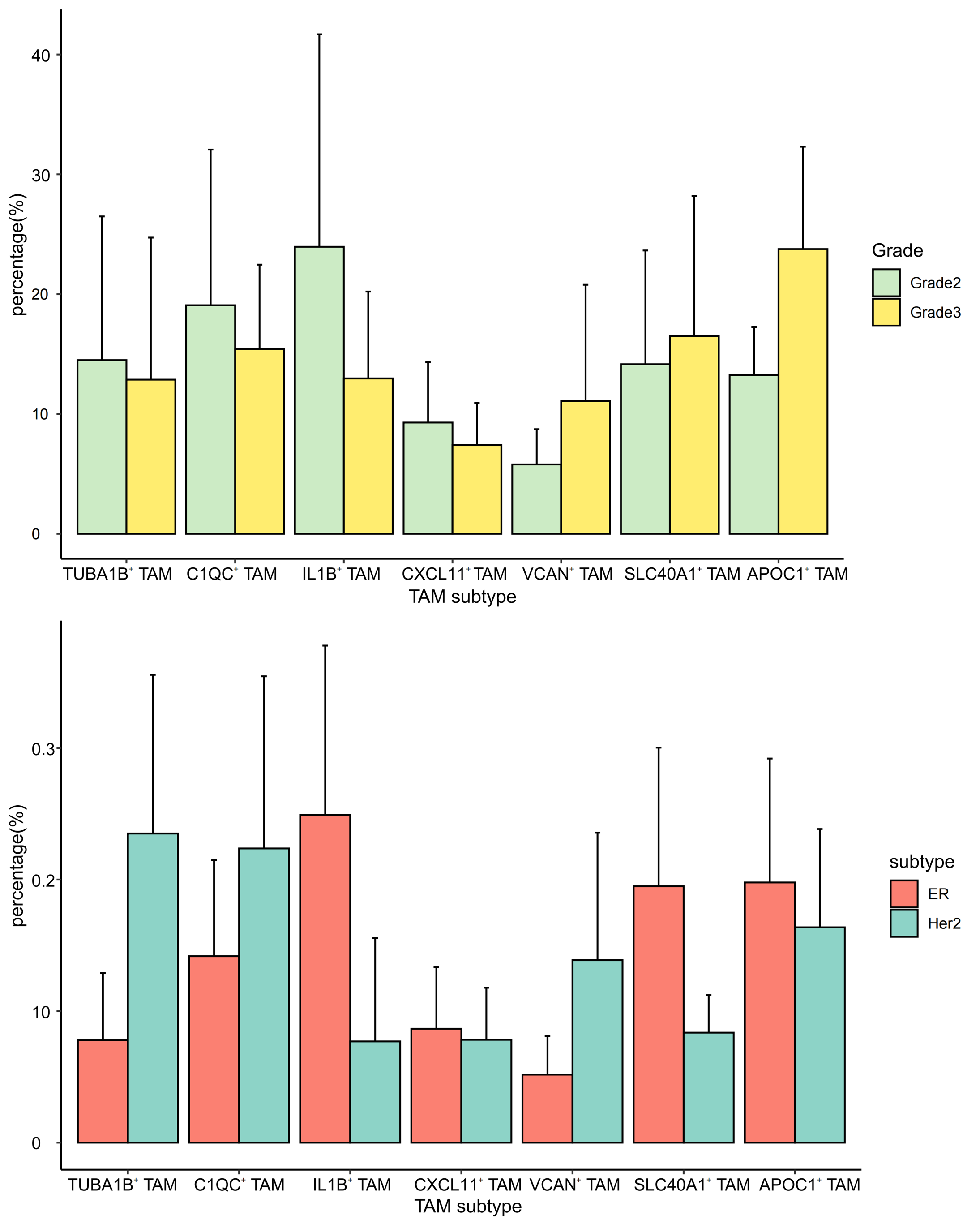

Supplement: Supplementary file 3 — Supplementary Material 3: Figure S3 Bar plots showing percentages of 7 TAMs to total TAMs in breast cancers of different grades (A) and subtypes (B) [file 13058_2024_1887_MOESM3_ESM.tif]

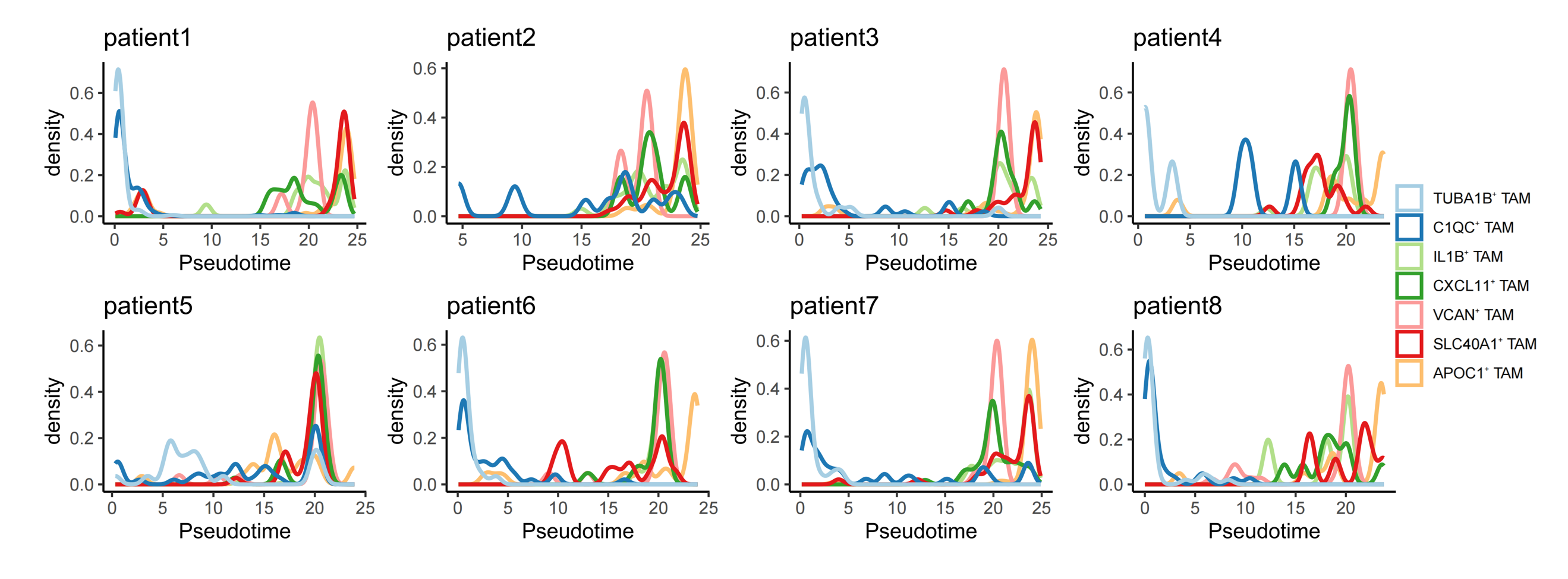

Supplement: Supplementary file 4 — Supplementary Material 4: Figure S4 The patterns of cell densities of 8 patients along with the pseudotime [file 13058_2024_1887_MOESM4_ESM.tif]

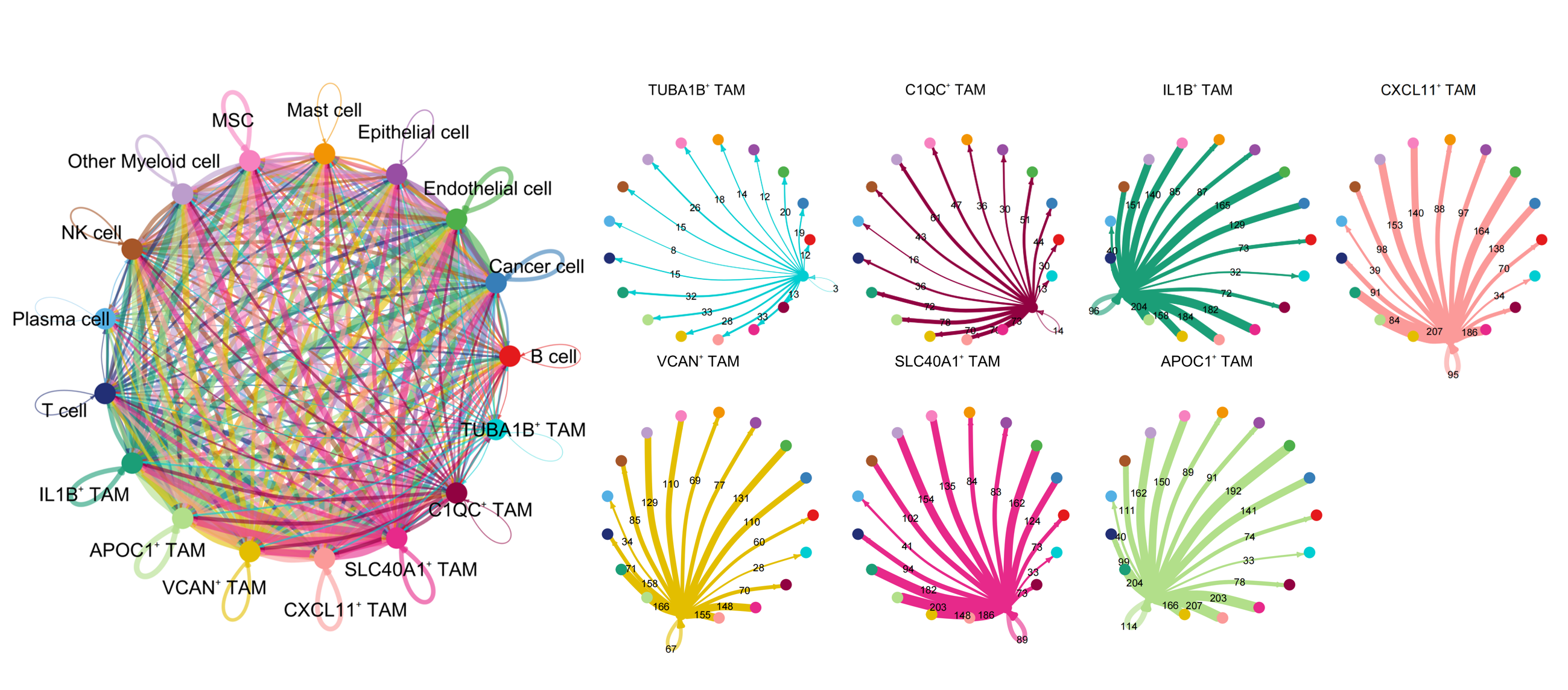

Supplement: Supplementary file 5 — Supplementary Material 5: Figure S5 Cell-cell interaction between TAMs and other cells in the TME. (A) Intercellular communication capacity between TAM and other cells in the TME. The color of each line indicates the ligand expressed by the cell population of the same color. Lines are linked to cell types that express homologous receptors. The thickness of the line is proportional to the number of ligands when homologous receptors are present in the receptor cell type. Loop lines indicate autocrine circuits. The figure quantifies potential communication but does not illustrate the anatomical location or boundaries of the cell types. (B) Details of ligands expressed by each major cell type and cells expressing homologous receptors to receive signals. Numbers indicate the number of ligand-receptor pairs connected between each cell [file 13058_2024_1887_MOESM5_ESM.tif]

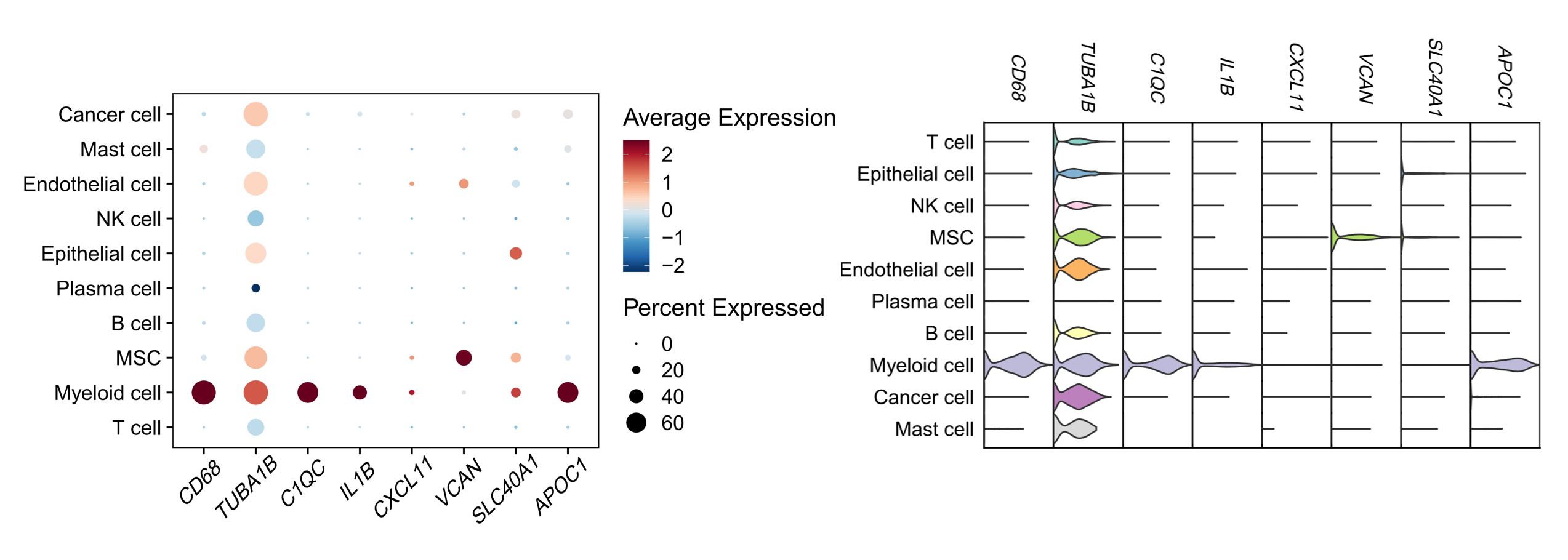

Supplement: Supplementary file 6 — Supplementary Material 6: Figure S6 Dot plot (A) and violin plot (B) showing expression levels of TAM subtype-specific genes (CD68, TUBA1B, C1QC, IL1B, CXCL11, VCAN, SLC40A1 and APOC1) across different main cell types in breast cancer [file 13058_2024_1887_MOESM6_ESM.tif]

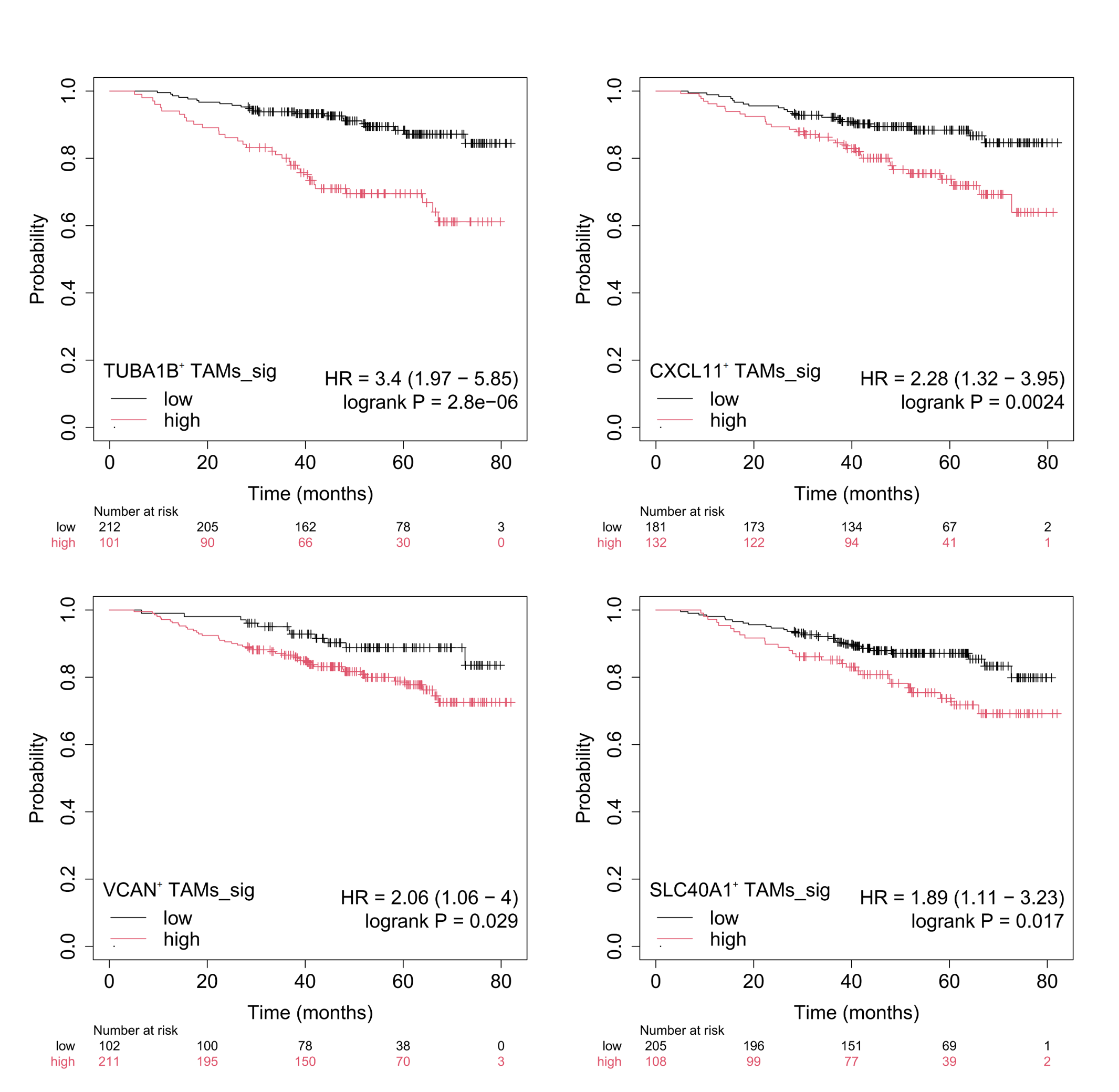

Supplement: Supplementary file 7 — Supplementary Material 7: Figure S7 Correlations of 4 subtypes of TAMs signature gene sets with OS. TUBA1B, HMGB1, TMSB10, TMSB4X for TUBA1B+ TAMs, CXCL11, CXCL10, CXCL9, ISG15 for CXCL11+ TAMs, VCAN, THBS1, S100A8, S100A9, FCN1 for VCAN+ TAMs and SLC40A1, FOLR2, CD163 for SLC40A1+ TAMs [file 13058_2024_1887_MOESM7_ESM.tif]
